# A ΣΔ Closed-Loop Interface for a MEMS Accelerometer with Digital Built-In Self-Test Function

**DOI:** 10.3390/mi9090444

**Published:** 2018-09-06

**Authors:** Dongliang Chen, Xiaowei Liu, Liang Yin, Yinhang Wang, Zhaohe Shi, Guorui Zhang

**Affiliations:** 1 MEMS Center, Harbin Institute of Technology, Harbin 150001, China; zoom_chen@126.com (D.C.); yinliang2003@126.com (L.Y.); 17B921023@stu.hit.edu.cn (Y.W.); smooth_nic@163.com (Z.S.); zgrhit@hotmail.com (G.Z.); 2 Key Laboratory of Micro-Systems and Micro-Structures Manufacturing, Harbin Institute of Technology, Harbin 150001, China; 3 State Key Laboratory of Urban Water Resource & Environment, Harbin Institute of Technology, Harbin 150001, China

**Keywords:** MEMS accelerometer, electromechanical delta-sigma, built-in self-test, in situ self-testing, digital resonator

## Abstract

Sigma-delta (ΣΔ) closed-loop operation is the best candidate for realizing the interface circuit of MEMS accelerometers. However, stability and reliability problems are still the main obstacles hindering its further development for high-end applications. In situ self-testing and calibration is an alternative way to solve these problems in the current process condition, and thus, has received a lot of attention in recent years. However, circuit methods for self-testing of ΣΔ closed-loop accelerometers are rarely reported. In this paper, we propose a fifth-order ΣΔ closed-loop interface for a capacitive MEMS accelerometer. The nonlinearity problem of the system is detailed discussed, the source of it is analyzed, and the solutions are given. Furthermore, a built-in self-test (BIST) unit is integrated on-chip for in situ self-testing of the loop distortion. In BIST mode, a digital electrostatic excitation is generated by an on-chip digital resonator, which is also ΣΔ modulated. By single-bit ΣΔ-modulation, the noise and linearity of excitation is effectively improved, and a higher detection level for distortion is easily achieved, as opposed to the physical excitation generated by the motion of laboratory equipment.

## 1. Introduction

In recent years, electromechanical sigma-delta (EM-ΣΔ) closed-loop MEMS accelerometer has been an active research field, due to its high-performance, inherent digital output, and convenience for post-processing. As the research on the EM-ΣΔ accelerometer has gone in-depth, it has demonstrated competitive performance compared to traditional macro-scale devices [1,2,3,4,5,6,7,8,9]. Previous research mainly focuses on the enhancement of noise performance [2,3,4] and the realization of requisite high-order ΣΔ architecture [5,6,7,8]. Such an accelerometer, with a noise floor as low as 200 ng/$\sqrt{\mathrm{Hz}}$ and a fifth-order EM-ΣΔ architecture, has already been reported [9].

Although the MEMS accelerometer has rapidly occupied the low-end commercial market with its high cost-performance, there are obstacles that limit its further development toward high-end applications (such as in aerospace and the military). In most of these situations, the accelerometer is often required to work in a harsh environment for a long period of time as a safety critical device. For these applications, the working reliability and performance stability are primary considerations [10]. Since any malfunction will induce disastrous consequences, any drift will be twice augmented by the integration, especially when performing long-term high-speed inertial navigation. However, due to the relatively large micromechanical manufacturing error, stress variation in material, surface effects of planar process, and fatigue of material [11,12], the MEMS-sensing element has a relatively poor long-term stability with respect to traditional macro devices.

Besides waiting for the evolution of MEMS process, the in situ self-test and self-calibration will provide a promising new point of view on these problems [13] and, thus, attract extensive research attention worldwide [13,14,15,16,17,18,19,20,21,22,23,24,25,26,27,28]. Although use of built-in self-test (BIST) units has been a routine technique in most mixed-signal system-on-chip (SoC) design flows [14,15], obstacles are encountered when implanting to an EM-ΣΔ system. This is due to the fact that the measurand of these systems is essentially physical, which creates difficulty with respect to precision using an electrical-only stimulus.

Direct implementation of electrostatic stimulus is only valid in a basic functional test, which aims to diagnose the defective dies in functional test or malfunction in practical usage. Many researchers have proposed diverse functional BIST methods, by incorporating the MEMS structure into a phase-lock loop (PLL) [16], resonator [17], or charge-pump [18] circuit, then, the working state of the circuit will be an indicator of malfunction. A more precise functional BIST is static symmetry testing, which can identify the location of defects by applying an electrostatic force on symmetrically distributed testing electrodes, and observing the output response [19]. However, the efficacy of these methods is limited, and the implementations are too dedicated to be widely adopted. 

In order to alleviate the difficulty in ensuring the precision and consistency of direct measurement, some researchers have resorted to indirect methods to realize the performance BIST. A widely adopted indirect test method is a so-called “alternate test”, which is first proposed by the engineers from TI Inc. for enhancing the test efficiency of analog ICs [20]. Recently, many researchers have worked on using this method to predict the key performance of MEMS sensors [21,22,23]. It is based on the principle that the mechanical performance undergoes the same environmental variations as the electrical performance; if the relationship between them can be precisely established, then the mechanical performance can be predicted by electrical test only. However, the process of establishing a precise mapping relationship needs a lot of repetitive work on sample collection and statistical analysis, which is time-consuming and can only be realized in a factory. 

Recently, some researchers have proposed a purely algorithmic method for calibrating the output of a 3-axis accelerometer [24,25,26,27,28]. It is based on the principle that in a static state, the vector sum of the 3-axis output should always be equal to the earth’s gravity. Based on this principle, a series of uncorrelated static measurements are performed. Then, the problem is shifted to the solving of a series of nonlinear multivariable equations. However, in this method, only linear drift error is taken into consideration, and the process of calibration does not utilize the cooperation of on-chip circuit, and the error inside the sensor still exists.

The EM-ΣΔ technique has many advantages compared to open-loop and analog closed-loop implementation [12,29].

Open-loop is a simple and cost-effective implementation which has been adopted in early designs. However, several drawbacks have been identified: since it is necessary to provide sufficient damping, more Brownian noise is introduced. Furthermore, the design latitude is constrained by the contradictory trade-offs introduced by the sensing element [12,29]. Since each element in the signal chain will add a distortion in final performance, the linearity of it is, thus, relatively poor.

As the research goes in depth, an analog closed-loop architecture has been proposed. As opposed to the open-loop system, the proof mass is well controlled at the equilibrium position by electrostatic feedback force. Thus, the linearity of the system is greatly improved. Since the electrical damping effect is introduced by the feedback force, the design trade-offs in the sensing element are released, and vacuum packaging technique can be used, resulting in a significant reduction in Brownian noise. Moreover, the bandwidth of the system has been significantly expanded by the feedback loop.

Recently, an EM-ΣΔ closed-loop architecture has been proposed, which incorporates the sensing element into a ΣΔ modulation loop. This configuration inherits the merits of analog closed-loop architecture, and has an additional advantage of direct digital output and compact architecture. Due to the time averaging effect of ΣΔ servo loop, the electrostatic feedback force is linearized, resulting in an improved linearity performance [1,2,3,4,5,6,7,8,9].

Thus, the EM-ΣΔ closed-loop accelerometer is the most advanced technique for building an interface circuit for accelerometers. However, the aforementioned methods either treat the sensing element as an individual device or treat the whole system as a black box. The BIST method, which is dedicated for this type of system, is rarely reported.

This paper has proposed a fifth-order EM-ΣΔ accelerometer with a digital built-in self-test function. The digital BIST circuitry is dedicated for the in situ dynamic distortion test of the EM-ΣΔ accelerometer. The traditional dynamic test method relies on the sophisticated vibration or shock machine [30], the inherent vibration distortion of which is usually large and limits the precision of the distortion test. The proposed BIST circuitry makes use of the ΣΔ modulated characteristic of the interface circuit. An on-chip 1-bit ΣΔ digital resonator is used to generate electrical excitation. Due to the noise reshaping nature of the ΣΔ loop, the in-band noise and distortion are well suppressed. The 1-bit signal has an inherently good linearity, and alleviates the need for a multi-bit multiplier. Thus, an area-efficient digital excitation source can be easily implemented on chip, as opposed to the difficulty in generation of analog or physical excitation.

This paper is organized as follows: Section 2 describes the system architecture and gives the theoretical analysis of the source of nonlinearity and the trade-offs between performance and stability. Section 3 gives the theoretical analysis and system level design of the BIST function. Section 4 gives implementation details and practical consideration of the proposed system. The experimental results are presented and discussed in Section 5, and the paper ends with conclusions in Section 6.

## 2. System Description and Topology Analysis

**Figure 1:**
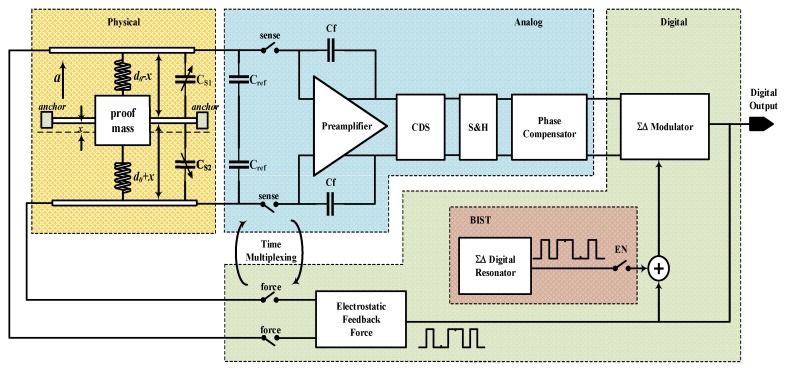
The block diagram of the whole electromechanical ΣΔ interface with built-in self-test (BIST) function.

The block diagram of proposed interface system is shown in Figure 1. A capacitive MEMS accelerometer is incorporated in a ΣΔ modulation loop with a third-order electrical integrator, constituting a fifth-order EM-ΣΔ system [5,31]. By time multiplexing technique, capacitance sensing and force feedback could be performed through the same sensing electrode, alternatively. This collocated sensing mechanism will simplify the design of sensing element and reduce higher-order resonance phenomenon [32]. The sensing element is configured as a balanced capacitive bridge with a pair of reference capacitors, thus, a fully differential architecture can be established. In the feed-forward path, there is a charge amplifier with a correlated-double sampling (CDS) function to realize the capacitance detection and reduce the low frequency noise. A phase compensator is inserted between the charge amplifier and electrical loop filter, in order to add some phase lead compensation to insure the loop stability. The BIST function is realized by an on-chip ΣΔ digital resonator. In BIST mode, a single-bit ΣΔ modulated sinusoidal wave will be injected into the digital part of the system under the control of external pin. As will be described in detail next, the output will be a reflection of the total harmonic distortion in the whole loop.

As evident, the electromechanical interface is a hybrid nonlinear feedback system comprised of components in different domains: physical, analog, and digital. Thus, comprehensive consideration should be taken at the beginning of the design stage.

### 2.1. Sensing Element

**Figure 2:**
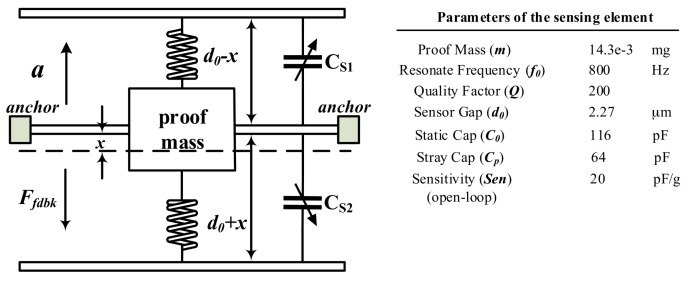
The mechanical model of the sensing element.

The sensing element is a critical part of the system which affects the stability, determines the sensitivity, and contributes to a major part of noise. A capacitive MEMS accelerometer is chosen as the front-end sensing element for its high output signal, low temperature sensitivity, and ease of applying electrostatic force to establish closed-loop control [33]. As shown in Figure 2, it generally consists of a proof mass suspended by cantilever beams anchored to a fixed frame and accompanied by a couple of fixed plates located on each side. 

By using Newton’s second law, the mechanical transfer function can be obtained:

(1) $$H_{ms}=\frac{x}{a}=\frac{1}{s^{2}+\frac{b}{m}s+\frac{k}{m}}=\frac{1}{s^{2}+\frac{\omega_{0}}{Q}s+\omega_{0}{}^{2}},$$

where *m* is the proof mass, *b* is the damping factor, *k* is the spring constant, $\omega_{0}=\sqrt{k/m}$ is the resonate frequency, $Q=\sqrt{km}/b$ is the quality factor. In closed-loop configuration, the response of the system is a comprehensive result of sensing element and interface circuit, and thus, the above parameters no longer dominate the system bandwidth, sensitivity, and resonance characteristic, therefore, more latitude can be obtained in choosing the mechanical parameters.

The Brownian noise caused by the motion of gas molecules and suspension beam is the major noise source in the mechanical part [12]. Since it is directly added into the front-end without any suppression, it will put a fundamental limit on the noise floor achievable and, thus, needs to be carefully treated. The Brownian noise equivalent acceleration (BNEA) can be expressed as:

(2) $$\mathrm{BNEA}=\frac{\sqrt{4k_{B}Tb}}{9.8m}\begin{array}{cc} & \left[g/\sqrt{Hz}\right] \end{array},$$

where *k_B_* is the Boltzmann constant, and *T* is the temperature in Kelvin. From the expression, we can find that a larger *m* and smaller *b* will help in reducing the intrinsic noise floor. Thus, most of the high-end MEMS accelerometers, including our design, use bulk micromachining technology and vacuum packaging technique to realize a larger proof mass and a smaller damping factor. The Brownian noise floor, in our design, is 17.4 $\mathrm{ng}/\sqrt{\mathrm{Hz}}$, calculated by the mechanical parameters used, thus, it is no longer a dominant noise source. However, the price paid here is that the mechanical part exhibits a highly underdamped response with a quality factor as high as 200, which makes insuring stability of the closed-loop a more challenging task. In our design, a phase-lead proportional differentiation (PD) controller is used as a phase compensator, but there are still trade-offs between stability and loop gain, which will be discussed next.

### 2.2. Electrostatic Feedback Force

For closed-loop operations, the system performance is mainly determined by the feedback path, as long as the loop gain is sufficiently large. In our system, the electrostatic feedback force is the only section in the feedback path, and the electrical BIST stimulus is converted to physical actuation force. Therefore, it has a significant effect on the key parameters of the system, including sensitivity, linearity, and dynamic range.

Consider, when a voltage drop *V* is imposed on either pair of sensing plates, there will be an attractive electrostatic force between them, which is given by

(3) $$F_{elec}=\frac{C_{0}d_{0}V^{2}}{2{\left(d_{0}+x\right)}^{2}},$$

where *C*_0_ is the static capacitance, *d*_0_ is the initial distance between the plates, and *x* is the displacement of proof mass at that time. There are two problems faced by force feedback:

- Nonlinearity: the electrostatic force is second-order related to voltage, and is modulated by the displacement *x*.
- Applying mechanism: the electrostatic force is always attractive, and there is normally no extra electrode for applying it, due to structural limitations, and the existence of high-order resonance mode [32,34].

**Figure 3:**
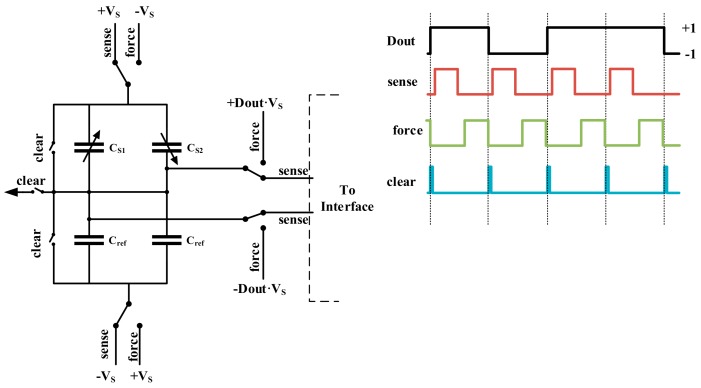
The diagram of ΣΔ time-multiplexed feedback mechanism.

One way to solve this problem is getting the linear result by subtracting a pair of balanced preload forces which are differentially changed on both sides, and realizing the frequency domain separation by modulating the measurand to high frequency [35,36,37]. However, there are interactions between sensing and force feedback mode, and if the preload force is above a certain limit, its polarity will reverse, resulting in instability [33,37]. Thus, in our system, we resort to oversampled ΣΔ force feedback to realize the linearization, and time-multiplexing technique to realize the separation of sensing and force feedback mode in the time domain. The applying mechanism of ΣΔ time-multiplexed feedback force is shown in Figure 3.

As shown, in each cycle, the front-end capacitive bridge is switching between two working phases: sense and force. In the sense phase, the bridge is biased, with supply voltage ±*Vs* and connected to back-end interface circuit. In the force phase, the proof mass is biased to negative supply, and the fixed electrodes are biased to either of the supply rails determined by the digital output signal *D_out_*. The two phases are working independently, and a clear phase is inserted to diminish the residue effect from the previous cycle, thus, the interaction effect is minimized. Since the oversampling frequency is well above the bandwidth of sensing element, it can be considered that the two phases are adding together at the same time.

The composite electrostatic feedback force subjected by the proof mass can be expressed as:

(4) $$F_{Feedback}=F_{P}-F_{N}=\frac{1}{2}\frac{C_{0}d_{0}{\left(-V_{S}-D_{out}V_{S}\right)}^{2}}{{\left(d_{0}+x\right)}^{2}}-\frac{1}{2}\frac{C_{0}d_{0}{\left(-V_{S}+D_{out}V_{S}\right)}^{2}}{{\left(d_{0}-x\right)}^{2}},$$

where *D_out_* is the digital output of the system which is quantized to ±1, and *x* is the residue displacement which is filtered by the second-order low pass characteristic of the sensing element. *F_P_* and *F_N_* are the electrostatic force imposed on positive side and negative side, respectively. It should be pointed out that Equation (4) is a general expression of composite electrostatic force which is effective at each sampling cycle. After a rearrangement, Equation (4) can be expressed as

(5) $$F_{Feedback}=\frac{\frac{1}{2}C_{0}d_{0}V_{s}{}^{2}[-4d_{0}x(1+D_{out}{}^{2})+4D_{out}(d_{0}{}^{2}+x^{2})]}{{\left(d_{0}{}^{2}-x^{2}\right)}^{2}}.$$

Note that the digital output *D_out_* is either 1 or −1, therefore, *D_out_*^2^ is always equal to 1 at each time point. Moreover, as the filtering characteristic of sensing element, only the low-frequency in-band part of the feedback force is effective, thus, consider the averaged feedback force:

(6) $$\bar{F_{Feedback}}=\frac{\frac{1}{2}C_{0}d_{0}V_{s}{}^{2}[-8d_{0}x+4\bar{D_{out}}(d_{0}{}^{2}+x^{2})]}{{\left(d_{0}{}^{2}-x^{2}\right)}^{2}}=\frac{F_{0}[-\frac{2x}{d_{0}}+\bar{D_{out}}(1+\frac{x^{2}}{d_{0}{}^{2}})]}{{\left(1-\frac{x^{2}}{d_{0}{}^{2}}\right)}^{2}},$$

where

(7) $$F_{0}=\frac{1}{2}\frac{C_{0}{\left(2V_{s}\right)}^{2}}{d_{0}}.$$

Equation (6) reveals that the average feedback force $\bar{F_{Feedback}}$ is first-order-related to the in-band output signal $\bar{D_{out}}$, and therefore, the relationship is linearized by a ΣΔ oversampling mechanism. On the other hand, it has to be said that there are first and second order modulation effects of displacement *x*, which will add additional distortion. If the loop gain is reasonably large, the condition of *x*^2^ << *d*_0_^2^ can hold, and Equation (6) can be rewritten as

(8) $$\bar{F_{Feedback}}\approx \bar{D_{out}}F_{0}-\frac{2x}{d_{0}}F_{0}.$$

There are only two first order terms: an ideal feedback force term with a linear displacement modulation term. Although the second term will add a feed forward path from displacement to feedback force introducing a gain reduction effect, the resulting system is still a first order system, and therefore, the nonlinear effect is diminished, in principle. Further increasing the loop gain, if the condition *x* << *d*_0_ holds, an ideal linear feedback system can be obtained. Thus, increasing the loop gain is the key to solving the distortion problem, but it will be obstructed by the stability problem, especially in a high-order ΣΔ system.

Besides introducing nonlinearity in feedback force, the displacement modulation effect will cause a more detrimental “pull-in” effect.

Consider when an electrostatic voltage is applied on one pair of parallel plates of the sensor, the equilibrium position of the proof mass can be found from the force-balance equation:

(9) $$\frac{C_{0}d_{0}V^{2}}{2{\left(d_{0}+x\right)}^{2}}=kx-f_{ext},$$

where *f_ext_* is the external force, which will introduce a zero-voltage gap of *x*_0_ = *d*_0_ − *f_ext_*/*k.* When the applied voltage is smaller than the pull-in voltage $V_{pi}=\sqrt{\frac{8kx_{0}{}^{3}}{27C_{0}d_{0}}}$ [38], the above function will have two solutions. As the voltage amplitude increases, when it exceeds *V_pi_*, no solution will be found and the system will collapse. Thus, the critical value of applied electrostatic voltage is *V_pi_*, which corresponds to an equilibrium position of 2*x*_0_/3. This effect comes from the fact that the electrostatic force is inversely proportional to the squared displacement *x*, while the elastic force is linearly proportional to the displacement *x*. As the displacement increases, the electrostatic force will increase faster than the elastic force introduced by cantilever beam, and after a certain limit, the equilibrium state will never establish, and the proof mass will collapse onto one of the static plates. This phenomenon will not only limit the usable range; once it happens, it may cause irreversible structure damage. 

The stability problem introduced by pull-in effect is a rather complicated problem, and it should be discussed with regard to different states [39,40,41].

For static state, the use of the proof mass is servo-controlled at the balanced place, as long as the input amplitude is within the representable range of the ΣΔ system. Thus, the static pull-in is well solved by the closed-loop control mechanism.

As for the dynamic “pull-in”, when the voltage changes quickly, the quasi-static regime does not apply. Both the damping forces and mass inertia need to be included in the model [42]. Furthermore, the case of the pull-in due to a step input and the pull-in due to modulated voltage is different, and warrants different treatment [43]. Our system is the modulated voltage case, in which the pull-in trigger point should be calculated from the accumulation effect of a series bits [43]. The dynamic pull-in is hard to be modeled, due to its strong nonlinear characteristics and because of the multiple solutions of the system state [39,44,45]. The modeling of the sensing element and stability analysis technique for multistate nonlinear systems need to be further researched, which is outside the scope of this paper.

### 2.3. ΣΔ Closed-Loop Interface

As mentioned, incorporating the sensing element in a ΣΔ closed-loop will release the design trade-offs faced by it, but the problem is shifted to the design stage of the back-end interface. Since the filtering ability of the sensing element is always insufficient for suppressing the quantization noise, the use of a high-order electrical filter in the succeeding interface circuit is a must, but will impair the stabilization of the closed-loop further as the quality factor of the front-end is made rather high, in consideration of Brownian noise. This section will be devoted to the design consideration and trade-offs with respect to these issues in the back-end interface circuit.

#### 2.3.1. Performance

**Figure 4:**
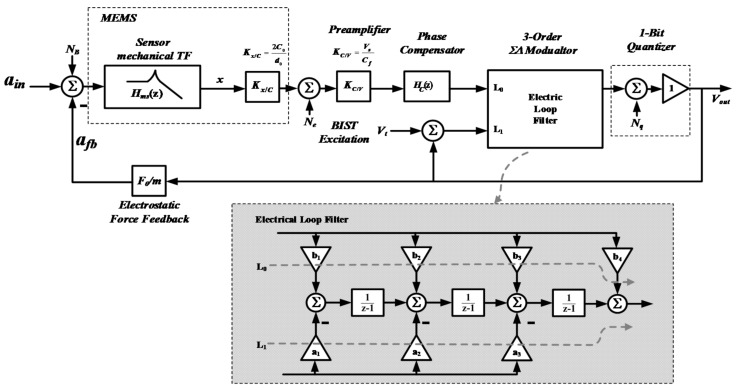
The mathematical abstracted model of the whole system.

The mathematical model of the whole system can be abstracted, as shown in Figure 4. Each element in Figure 1 is expressed by its mathematical function. 

The sensing element is expressed with its transfer function *H_ms_*(*z*) in z domain, followed by a gain stage *K_x_*_/*C*_ to translate it to capacitance change. The transformation from *H_ms_*(*s*) in s domain to *H_ms_*(*z*) in *z* domain, taking time-multiplexed effect into consideration, is derived as detailed in paper [46], and thus, is not discussed in this paper. The only conclusion needed to be cited here is that the use of time-multiplexing only introduces a gain loss and a duty-cycle-related time delay. Besides that *H_ms_*(*z*) still exhibits a second order filtering characteristic. The front-end pre-amplifier is abstracted as a gain stage *K_C_*_/*V*_, and the phase compensator is expressed as its *z*-form expression *Hc*(*z*). To provide a direct and concise viewpoint, the 3-order ΣΔ modulator is expressed by its feed-forward path transfer function *L*_0_, and feedback path transfer function *L*_1_ [47]. For the distributed feedback topology we take, *L*_0_ and *L*_1_ is given by

(10) $$L_{0}(z)=\sum_{i=1}^{4}b_{i}{\left(\frac{1}{z-1}\right)}^{4-i},$$

(11) $$L_{1}(z)=-\sum_{i=1}^{3}a_{i}{\left(\frac{1}{z-1}\right)}^{3-i}.$$

Then, the signal transfer function *STF*_3-*order*_ and noise transfer function *NTF*_3-*order*_ of the 3-order electrical ΣΔ modulator can be expressed by

(12) $$STF_{3-order}(z)=\frac{L_{0}(z)}{1-L_{1}(z)},$$

(13) $$NTF_{3-order}(z)=\frac{1}{1-L_{1}(z)}.$$

Before discussing the influence factors of the performance of EM-ΣΔ loop, it should be noted here that the two major sources of performance degradation, quantization noise and residue displacement, undergo different loop processing; thus, they should be discussed separately.

**Figure 5:**
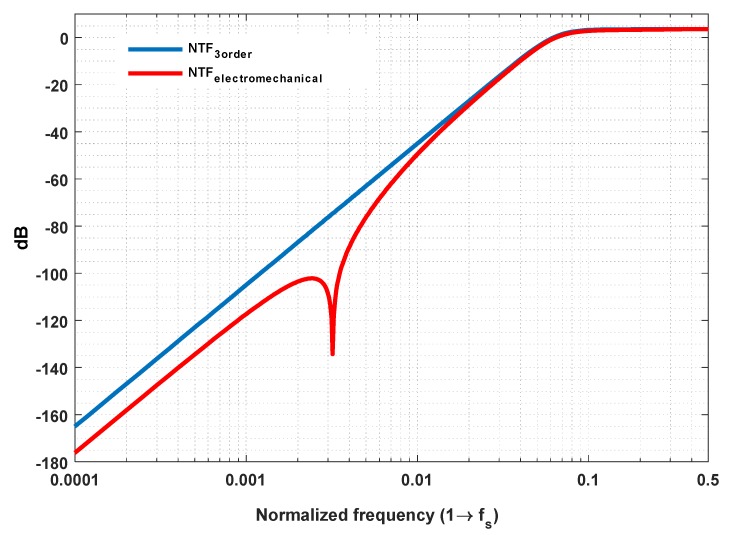
Spectrum of equivalent output quantization noise.

First, consider the quantization noise. After sufficiently suppressing Brownian noise, the quantization noise will be the major limitation of the noise floor achievable. It is added into the loop at the quantizer node, and the noise transfer function *NTF_EM_* from quantization noise to output can be derived by analyzing the mathematical model shown in Figure 4:

(14) $$NTF_{EM}(z)=\frac{1}{1-L_{1}(z)-L_{0}(z)G(z)},$$

(15) $$G(z)=\frac{F_{0}K_{x/C}K_{C/V}H_{ms}(z)H_{C}(z)}{m},$$

where *G*(*z*) is the overall transfer function of the mechanical branch. The denominator of Equation (10) indicates that the quantizer noise is suppressed by a composite filtering effect provided by a 3-order term *L*_1_ and a 5-order term *L*_0_*G*. However, it should be noted that the overall noise performance is inferior to a 5-order electrical ΣΔ modulator. This is due to the fact that, as opposed to a 2-order electrical integer, the in-band gain of the mechanical branch is flat and limited, moreover, it will be weakened by the use of time-multiplexing technique and phase compensator. Thus, the efficacy of quantization noise suppression is mainly determined by the inside 3-order electrical integer, as shown in Figure 5. Therefore, for our system, in order to achieve sub-μg/$\sqrt{\mathrm{Hz}}$ noise performance, a 3-order inside electrical modulator is necessary.

Besides noise, linearity is another key performance parameter which often imposes an upper limit on the range of signal amplitude that can be precisely represented. As mentioned in Equation (6), in our system, the main source of non-linearity comes from the fact that the feedback electrostatic force has a nonlinear displacement modulation effect, since the square-law effect of it is linearized by the use of ΣΔ modulation. For a closed-loop operation, the most straightforward way to suppress the residue displacement is to increase the loop gain seen by it. However, note that, if the loop is breaking at different node, the effective loop gain is different. As a result, although the loop gain seen by quantization noise is sufficiently high, this is not the case for residue displacement. When breaking at the displacement node, the inside 3-order modulator should be considered as a whole, thus, the loop gain seen by displacement *x* can be expressed as

(16) $$G_{x}(z)=\frac{F_{0}K_{x/C}K_{C/V}H_{ms}(z)H_{C}(z)STF_{3-order}(z)}{m}.$$

**Figure 6:**
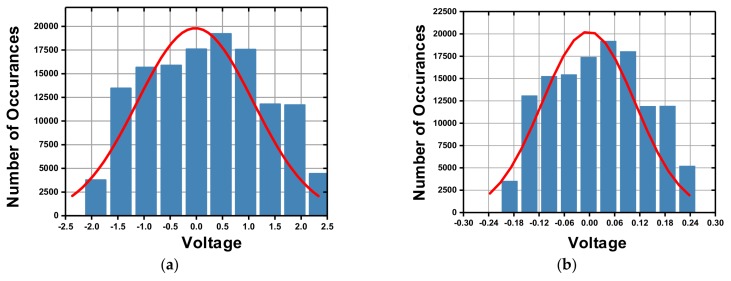
Distribution plot of the amplitude in front of quantizer: (**a**) loop gain enhanced by reinforcing the feed-forward path; (**b**) loop-gain enhanced by scaling of the feedback path *L*_1_.

As evident, the 3-order integrating effect, which is the major force in gain enhancement, is missing in this expression. As a result, the loop gain seen by residue displacement is much less than that seen by quantization noise. In order to make up for this loss, an additional gain stage is needed. However, if it is inserted in the feed-forward path, there will be more energy appearing in front of the quantizer, which will result in an earlier saturation. An alternative way is to scale the coefficient in the feedback path *L*_1_, which can easily be realized by scaling the feedback voltage. As shown in Equation (11), the decrease of *L*_1_ will give rise to *STF*_3*-order*_. The distribution plot of the input voltage in front of quantizer is shown in Figure 6. As shown, the scaling of *L*_1_ will also help in reducing the signal amplitude in the feed-forward path, which will make the behavior of the electrical circuit more ideal. However, we must admit that the decreasing of *L*_1_ will cause a reduction in the quantization noise suppressing ability due to the reduced loop gain. Besides that, there will need more continuous logic levels to draw the input back, which means more susceptible to instability. In our system, *L*_1_ is scaled as one-tenth of *L*_0_, to make a compromise to those trade-offs.

#### 2.3.2. Stability

**Figure 7:**
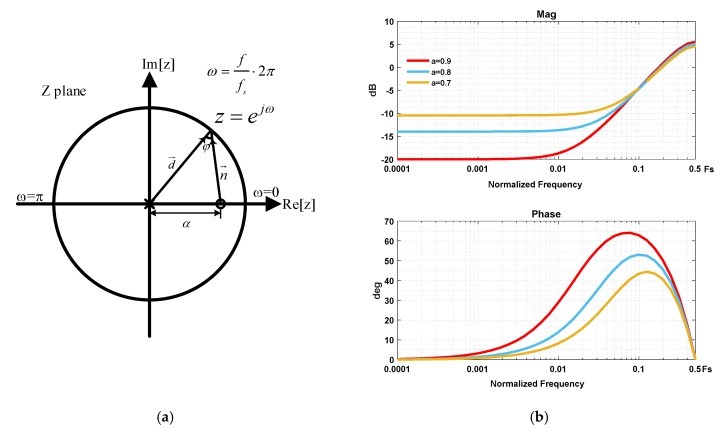
The frequency response of phase compensator: (**a**) the poles and zeros distribution; (**b**) the magnitude and phase response.

Next, we come to the stability problem. In order to maintain stability, a phase compensator is inserted in the feed-forward path, whose expression is given by

(17) $$H_{C}(z)=\frac{z-\alpha }{z},$$

where *α* is a compensation factor whose value is in the range of (0, 1). The distribution plot of poles and zeros is shown in Figure 7a. The frequency response of *H_C_*(*z*) can be found by graphically considering the magnitude and phase of vectors connecting its poles and zeros to point around the unit circle. As evident, the magnitude is minimum at dc, and starts increasing until the frequency reaches 0.5*f_S_*, resulting a limited variation from 1 − *α* to 1 + *α.* Also, there is a positive phase angle *φ* varying with frequency, which starts from zero at dc, and reaches its maximum when vector $\vec{n}$ is perpendicular to the real axis, and falls to zero again when the frequency reaches 0.5*f_S_*. Thus, the name phase compensator [9] is derived from the fact that the gain variation is limited and concentrated around dc, but there is a positive phase lead which will exert its influence at crucial frequencies. The bode plot of phase compensator is shown in Figure 7b.

It can be found that a larger compensation factor will give a better phase compensation result, but at the expense of a larger gain loss in low frequency. An insufficient in-band loop gain means a reduction in quantization noise suppression and a larger residue displacement. Apart from the compensation factor, the sampling frequency is another factor influencing the result. A proper sampling frequency is preferred at which the phase lead is maximum, but it means that the sampling frequency cannot be chosen too high, in which case, the phase lead is not exerting its influence yet, which is also contradictory to performance consideration. Thus, the use of phase compensator is a compromise of performance and stability, the parameter of which should be carefully identified through a number of simulations.

**Figure 8:**
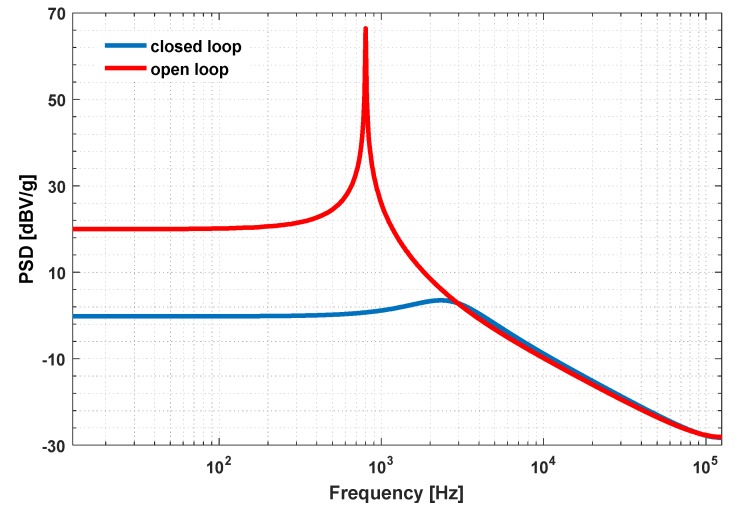
Compensated closed-loop response vs open-loop response.

The power spectrum density (PSD) plot of the compensated closed-loop is compared to that of open-loop configuration in Figure 8. As shown, the use of closed-loop configuration has extended the bandwidth, and the resonating peak is flattened by the use of phase compensator.

## 3. BIST Function

In order to provide a cost-effective way for in situ self-test of harmonic distortion, a purely digital BIST circuitry is embedded in the system. There are two major challenges of designing a BIST circuitry for an accelerometer.

First is selecting the entrance of BIST stimulus. This is due to the fact that the sensing element is part of ΣΔ closed-loop, which could not be disconnected to impose electrostatic force, and extra driving electrode is not available in the general case. Thus, we should find an inner node in the feedback loop to apply the electrostatic force indirectly. The principle of entrance selection is that the applying point and observing point should cross over the sensing element, if not, the observed response will not reflect the characteristic of the sensing element. For example, if the BIST stimulus is directly added into the output node, due to the control of closed-loop, the output voltage will always equal to the input stimulus as long as the loop gain is sufficiently high, irrespective of any change in sensing element. In the proposed method, we choose the feedback point of the 3-order electrical filter as the entrance of BIST stimulus. The signal at this point is 1-bit quantized, thus, the use of digital excitation is convenient. 

Consider that the BIST stimulus is a 1-bit ΣΔ modulated signal *V_t_*. Then, by calculating the linear module shown in Figure 4, the transfer function from *V_t_* to *V_out_* can be expressed as

(18) $$\frac{V_{out}}{V_{t}}=\frac{L_{1}(z)}{1-L_{1}(z)-L_{0}(z)G(z)},$$

which can be rearranged as

(19) $$\frac{V_{out}}{V_{t}}=\frac{\left(\frac{L_{1}(z)}{1-L_{1}(z)}\right)}{1-\left(\frac{L_{0}(z)}{1-L_{1}(z)}\right)G(z)}.$$

For in-band test signal *V_t_*, the condition *L*_1_(*z*) >> 1 can hold, and by using Equation (12), the Equation (19) can be rewritten as

(20) $$\frac{V_{out}}{V_{t}}\approx \frac{1}{1-G(z)STF_{3-order}(z)}=\frac{1}{1-G_{x}(z)}\approx -\frac{1}{G_{x}(z)}.$$

It can be found that the transfer function from *V_t_* to *V_out_* is the reverse of loop gain seen by residue displacement, which is the major determinant of the harmonic distortion of the whole loop. Thus, the value of loop gain and the harmonic distortion level can be determined by performing a single tone BIST test and observing the output response.

The other challenge that should be dealt with is to implement an embedded high-precision digital excitation source cost-effectively. There are multiple implementation methods of the digital excitation source, like direct digital synthesizing (DDS) or fixed-length recording technique [48]. However, the first method requires a large amount of hardware resources, and the second is lack of flexibility in signal control, thus, both of them are not suitable for implementation of on-chip BIST circuitry. 

**Figure 9:**
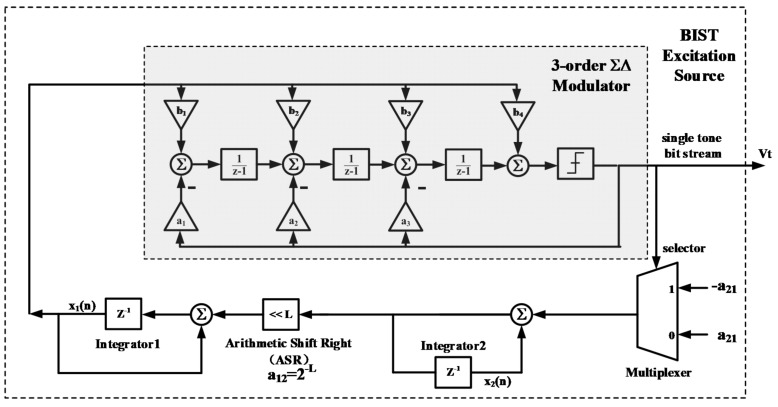
Block diagram of BIST excitation source.

The BIST excitation source in our design is implemented using the ΣΔ resonating circuitry proposed in [49], and its block diagram is shown in Figure 9. The resonator incorporates a ΣΔ modulator in the feedback loop, resulting in concise architecture with an inherent 1-bit ΣΔ modulated output and no need for an area-consuming multibit multiplier. In order to enhance the signal to noise & distortion ratio (SNDR) performance to meet the BIST requirement, the ΣΔ modulator is implemented using 3-order cascode of integrators feedback (CIFB) architecture. The STF of it is set to 1 in order to minimize the introduced phase delay and simplify the selection of loop coefficient *a*_12_ and *a*_21_.

For in-band signal, the loop gain of the resonator can be expressed as

(21) $$G_{R}(z)=\frac{-a_{12}a_{21}z^{-1}STF_{3-order}}{{\left(1-z^{-1}\right)}^{2}}.$$

In order to keep a stable oscillation, according to the Barkhausen criterion, *G_R_*(*z*) should be equal to 1. The characteristic equation of the BIST stimulus generator becomes

(22) $$z^{-2}+(a_{12}a_{21}STF_{3-order}-2)z^{-1}+1=0.$$

For an in-band signal, *STF*_4*-order*_ can be considered as a constant equal to 1. To simplify the control mechanism, we choose 0 < *a*_12_*a*_21_ < 2, and the resonating frequency can be deduced:

(23) $$\omega_{0}=f_{S}\cos^{-1}(1-\frac{a_{12}a_{21}}{2}),$$

where *f_S_ =* 1*/T_s_* is the sampling frequency of the system. It can be found that the resonating frequency is determined by the product of *a*_12_ and *a*_21_. So far, only the in-band signal is concerned when performing above analysis. However, there is a quantization noise part which will inject additional energy into the loop and make the oscillation unstable. Thus, the value of *a*_12_ and *a*_21_ are chosen to be much less than 1, to alleviate this injection. This will limit the resonating frequency to a relatively low value, but is just suitable for a harmonic test.

Besides frequency, the amplitude of oscillation can be controlled by choosing the initial condition of the loop integrator, denoting the values of registers in integer1 and integer2 to be *x*_1_(n) and *x*_2_(n). For simplicity, the initial value *x*_2_(0) is chosen to be zero. The value of *x*_1_(n) at time node n = 0 and n = 1 can be expressed as

(24) $$x_{1}(0)=A\sin(\phi ),$$

(25) $$x_{1}(1)=A\sin(\omega_{0}T_{s}+\phi ).$$

Using one iteration calculated from the model shown in Figure 9, note *x*_2_(0) = 0, the relationship of *x*_1_(0) and *x*_1_(1) can be obtained:

(26) $$\frac{x_{1}(1)}{x_{1}(0)}=\frac{A\sin(\omega_{0}T_{s}+\phi )}{A\sin(\phi )}=1-a_{12}a_{21}$$

Expanding Equation (26), we can obtain

(27) $$\cot(\phi )=\frac{1-a_{12}a_{21}-\cos\omega_{0}T_{s}}{\sin\omega_{0}T_{s}}.$$

Using the relationship shown in Equation (23), and for oversampling systems, the condition *ω*_0_*T_s_* << 1 can hold, then we can obtain

(28) $$\cot(\phi )={\frac{\cos\omega_{0}T_{s}-1}{\sin\omega_{0}T_{s}}|}_{\omega_{0}T_{s}\to 0}=0.$$

Then, the amplitude *A* and initial phase *Φ* of the oscillation can be expressed:

(29) $$\left\{ \begin{array}{l} A=x_{1}(0)\\ \phi =\frac{\pi }{2} \end{array}\right.$$

In conclusion, the characteristic of BIST stimulus can be controlled by tuning the loop parameters, (e.g., tuning *a*_12_ and *a*_21_ for frequency control and tuning *x*_1_(0) for amplitude control). In order to avoid the using of multibit multiplier, *a*_12_ is set to 2^−L^ and realized by an arithmetic shifter to the right (ASR), resulting in a coarse frequency tuning. The value *a*_21_ and *x*_1_(0) is restored in on-chip registers that can be selected through a reserved digital interface for realizing a flexible test strategy.

## 4. Circuit Implementation Details

**Figure 10:**
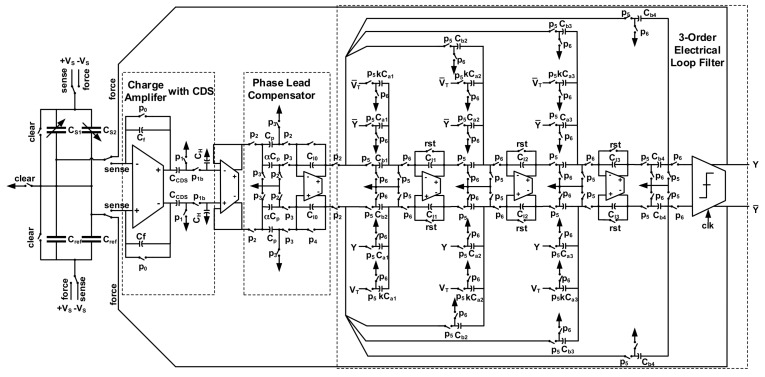
The schematic of proposed EM-ΣΔ accelerometer.

The schematic of proposed EM-ΣΔ accelerometer is shown in Figure 10. The system model shown in Figure 4 is implemented with switch capacitor (SC) circuit. 

In the first stage, the capacitance change is converted to voltage by a charge amplifier. It has adopted an output correlated double sampling (CDS) technique, thus, low frequency in-band noise and dc offset from front-end amplifier are greatly reduced. The phase lead compensator is implemented using a summing amplifier. By summing the charge corresponding to no-delay in-phase signal, and delayed out-of-phase signal together, the transfer function shown in Equation (17) can be realized. The 3-order electrical loop filter is implemented by also using an SC circuit. In order to prevent the system from locking into saturation state in practical condition, a reset signal is added to clear the integrating capacitors, if needed. The BIST circuitry is implemented with verilog code, and synthesized to layout with electronics design automation (EDA) tools, and thus, is not shown in this schematic. The stimulus *V_T_* generated by BIST circuitry is added into the loop through a replicated feedback path, and thus, the addition with system output is realized indirectly.

**Figure 11:**
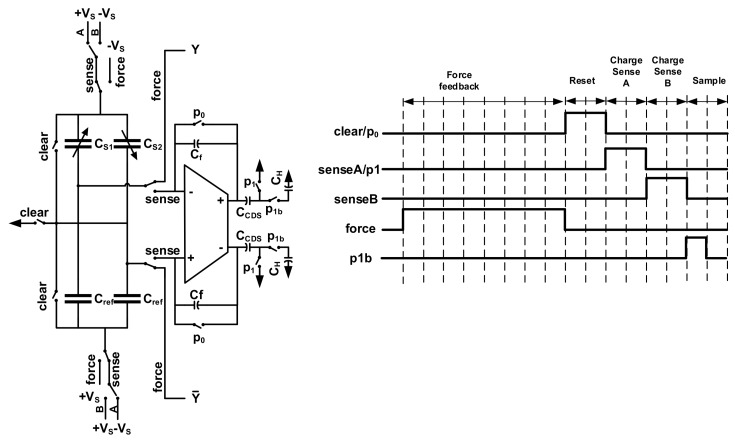
The schematic of front-end charge amplifier with CDS function.

In order to remove the low frequency noise and offset in the front-end amplifier, the CDS technique is used. The circuit implementation and clock diagram are shown in Figure 11. At reset phase, each electrode of the sensing element is connected to the ground, in order to diminish the residue effect of previous feedback force. The charge on the feedback capacitor C_f_ is discharged too. Next, at phase A, the sensing element is charged by a pair of supply voltages, and the differential capacitance change is calculated by the SC circuit and stored on the capacitor C_CDS_. After that, in phase B, the polarity of the charge voltage of the sensing element is inverted, inducing a negative voltage change at the output of charge amplifier. Before that, C_CDS_ is disconnected from the GND, leaving the node floating. Thus, after phase B, the output results perform a subtraction operation on the capacitor. Note that, the relative polarity of the low frequency noise and offset does not change, thus, they canceled each other out, due to the CDS operation. After that, the voltage at the floating node of C_CDS_ is only related to the measurand, and is sampled on the hold capacitor C_H_ at the next sampling phase.

**Figure 12:**
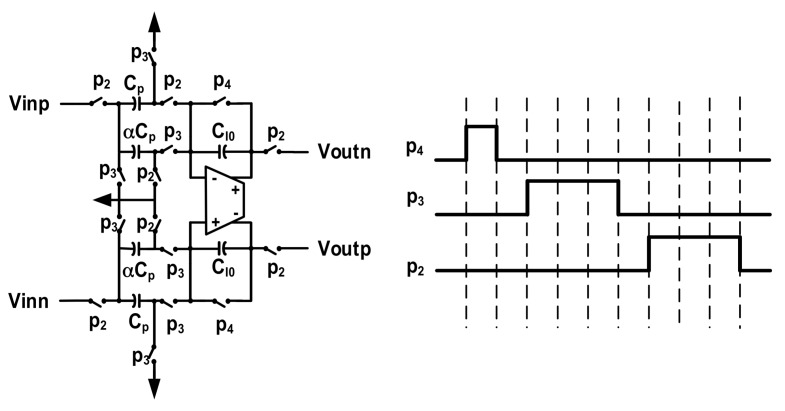
The schematic and timing diagram of phase compensator.

As mentioned, in order to reduce the Brownian noise, the sensing element is sealed in a vacuum package, resulting in a highly underdamped frequency response. When the sensing element is incorporated into a closed-loop, a phase compensator with a transfer function 1 − *αz*^−1^ should be added to provide some phase lead. The transfer function is realized using the SC circuit shown in Figure 12. In each cycle, the charge of previous cycle restored on C_I0_ is first cleared by p_4_. In the next phase, p3, the inverse charge of previous cycle restored on αC_p_ is pushed onto the integrating capacitor C_I0_, and the charge on C_p_ is cleared. Then, at phase p2, the input value of current cycle is sampled on C_p_ and transferred on to C_I0_ at the same time. Thus, at that time, the output value is the sum of the charge restored at previous cycle and the charge sampled at current cycle.

Then, the transfer function of this section can be written as

(30) $$H_{c}(z)=\frac{C_{p}}{C_{I0}}-\alpha \frac{C_{p}}{C_{I0}}z^{-1}.$$

Normally, we choose C_p_ = C_I0_, then the above function is the same as Equation (17). The ratio, *α*, of the sampling capacitor will set the compensation depth.

**Figure 13:**
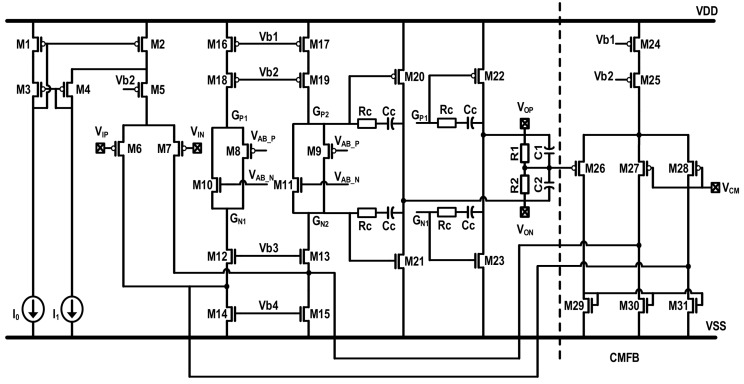
The schematic of proposed EM-ΣΔ accelerometer.

The schematic of the OTA used in proposed system is shown in Figure 13. In order to reduce the harmonic distortion, second stage with class AB output architecture has been used. A regulated cascade current source (M1~M5) is implemented as the tail current of input stage, in order to enhance impedance of it, and hence, the common mode and supply rejection performance. The common mode feedback circuit is using a parallel RC detector with an auxiliary amplifier, in order to enhance the working range and the common mode loop gain. It should be noted here that the parasitic capacitance of the sensing element will be charged and discharged at the same time, which will require additional current output ability and bandwidth margin. Thus, the front-end charge amplifier should be especially powerful and high-speed. Due to the use of two stage class-ab architecture, a 100 mA peak output current and 100 MHz gain bandwidth production (GBW) is easily obtained. The remaining amplifiers in Figure 10 are a scaled version of this architecture. 

## 5. Results and Discussion

The complete electromechanical ΣΔ closed-loop interface circuit with BIST function is implemented using 0.35 μm CMOS BCD process. The interface chip contains switch-capacitor circuits as the analog part, and on-chip timing sequence and BIST circuit as the digital part. The reference clock is 2 MHz, which is generated by an off-chip crystal oscillator to obtain a stable timing reference. After on-chip phase adjustment, a sampling clock at 250 kHz is used as the main clock. The total area of the ASIC is 11.2 mm^2^. The area of BIST circuit is 0.86 mm^2^, and only occupies 1/13 of total area. And the power dissipation is 32 mW with a ±2.5 V supply voltage. 

**Figure 14:**
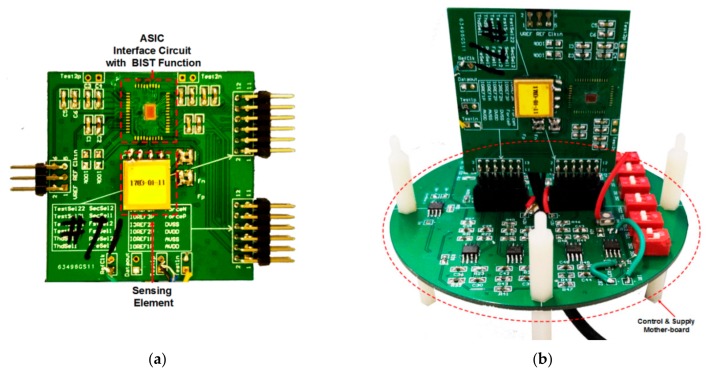
The photograph of prototype test board. (**a**) The daughter board with ASIC and sensing element; (**b**) The motherboard with the daughter board mounted.

The prototype test board is shown in Figure 14. It is composed of a motherboard with supply regulator and control logic, and a daughter board with ASIC and sensing element. The sensing element is sealed in a vacuum ceramic package and connected to the ASIC on a print-circuit-board (PCB) board. On the daughter board, a pair of matching capacitors is needed to build the balanced capacitive bridge with the sensing element, and provide some calibration ability of the imbalance between the two sensing capacitors. The amplitude and frequency of on-chip BIST stimulus can be controlled by the logic signal generated on the motherboard, which will select one of the pre-load voltages stored in the registers, whereby the connector on the PCB and the reserved interface in the ASIC.

**Figure 15:**
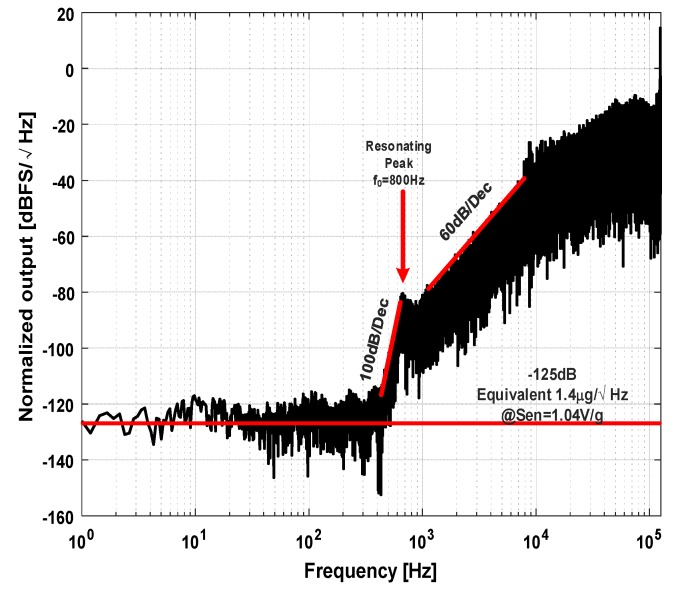
Normalized output noise spectrum at 0 g condition.

The output bit stream of the system and BIST stimulus are brought out through shielded wires and captured by a logic analyzer. The calculated PSD of the output noise spectrum at static 0 g condition is shown in Figure 15. The PSD is normalized to a full-scale voltage of 2.5 V. The noise floor of the system is about −125 dB, which is equivalent to an input acceleration noise of 1.4 μg/$\sqrt{\mathrm{Hz}}$ (with a measured sensitivity of 1.04 V/g). From Figure 13, an evident resonating peak due to the underdamping characteristic of the sensing element can be found. Before that frequency, the spectrum shows a fifth-order noise-shaping characteristic, and after that frequency, the noise-shaping characteristic degrades to a three-order one, which is consistent with the aforementioned analysis.

**Figure 16:**
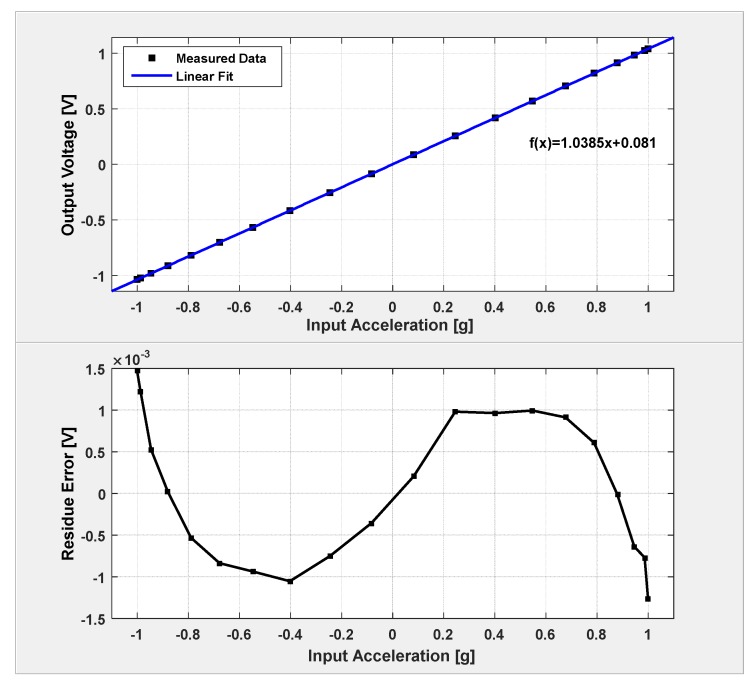
Static sensitivity and linearity results.

The static sensitivity and linearity of the system is identified by a series of static tests at different orientation in gravitational field. The prototype board is perpendicularly mounted on a dividing head, and the input gravitational acceleration can be calculated by the rotation angle readings. The static sensitivity and linearity test result is shown in Figure 16. As shown, the sensitivity of the closed-loop system is 1.04 V/g, and the static linearity is 0.708%. 

**Figure 17:**
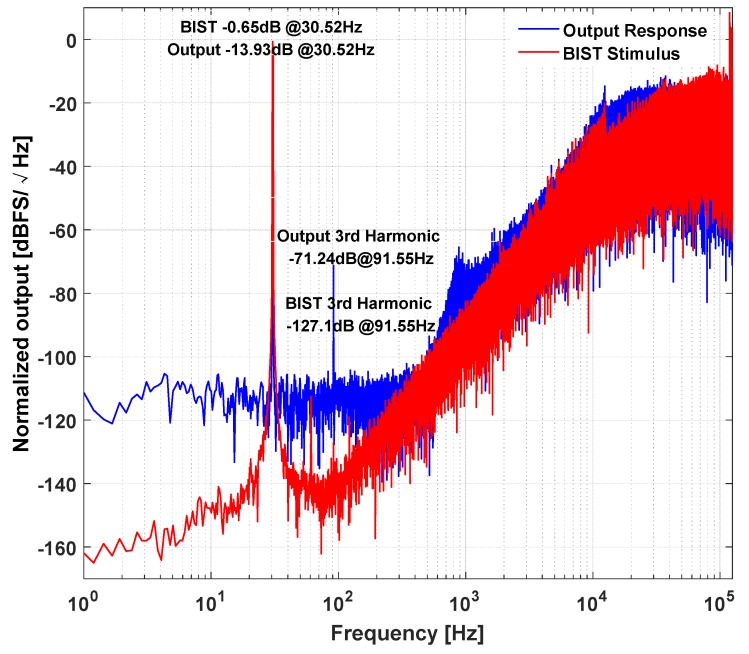
The power spectrum density (PSD) of the BIST stimulus and the output response induced by it.

The harmonic distortion test is fulfilled by the on-chip BIST function. The sensing element is oriented at 0 g condition. A 1-bit ΣΔ modulated sinusoidal wave is generated by on-chip BIST resonator and injected into the loop to excite the proof mass to a constant oscillation. The amplitude and frequency of the BIST stimulus is set by the logic control signal generated on the mother board. Both the BIST stimulus and induced output bitstream is captured by a logic analyzer. Their calculated PSD is shown in Figure 17. It can be found that the noise and harmonic distortion of the BIST stimulus is well controlled by digital ΣΔ modulation technique. Furthermore, the noise floor of the output response in BIST test is slightly higher that of static test. This is due to the fact that the in-band quantization noise in the BIST stimulus is still injected into the loop, although most of it is suppressed by ΣΔ modulation. The third order harmonic of the BIST stimulus is −127 dB, while the third order harmonic measured in the output is −71 dB. This means that the loop gain is not sufficiently high, and the residue displacement does not get sufficient suppression, thus resulting in an obvious 3-order harmonic distortion. As shown in Equation (19), the loop gain of the closed feedback loop can be read by the ratio of BIST amplitude and output amplitude. By calculating the power of the signal bins, the amplitude ratio of BIST and output signal can be obtained, then, the loop gain is calculated as 13.95. As shown in Section 2.3, the feedback voltage of the 3-order electrical filter could give some latitude for gain enhancement.

**Figure 18:**
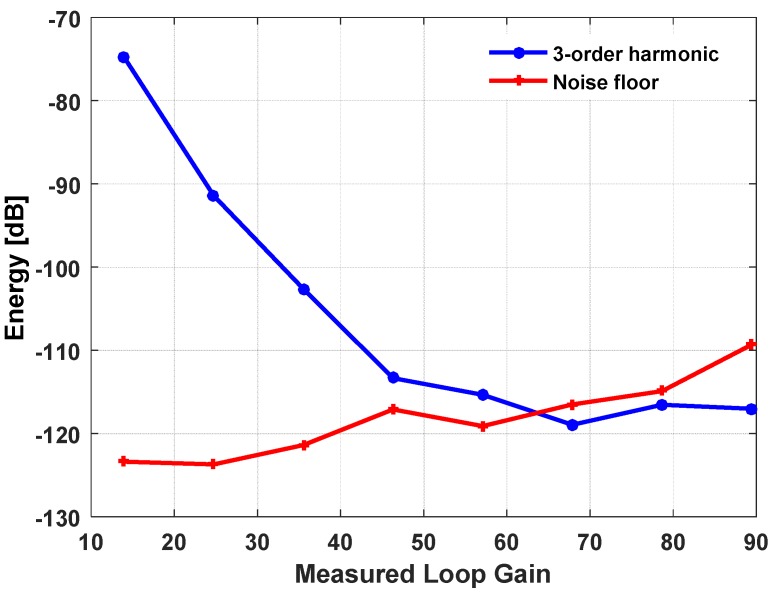
The relationship between the measured loop gain vs 3-order harmonic and output noise floor.

The feedback voltage of the 3-order electrical filter is preserved for off-chip application. It is generated by the voltage regulators on the mother board. A series of BIST tests are carried out under different loop gain by adjusting the feedback voltage. The relationship between the measured loop gain, the harmonic distortion, and the noise floor is shown in Figure 18. It can be seen that as the loop gain increases, the harmonic distortion is effectively suppressed. However, the noise floor of the output will have a tendency to rise up. This is due to the fact that the scale of the feedback voltage will introduce an energy increase in front of the quantizer, and hence, equivalently decrease the loop gain, thus resulting in a relatively low noise suppressing ability. Both of the abovementioned two tendencies determine the minimum detectable harmonic distortion, since, when the noise floor rises close to the harmonic distortion level, the readings will not be accurate representations. As shown, the minimum securely detectable harmonic distortion is about −110 dB.

## 6. Conclusions

This paper has presented the design of a high-order electromechanical ΣΔ interface chip for a high-Q capacitive MEMS accelerometer. Most of our attention has been put on the loop nonlinearity. The source of nonlinearity is analyzed in detail, and we point out that after the linearizing effect provided by 1-bit ΣΔ modulation, the main source of nonlinearity comes from the residue displacement modulation effect. As we analyzed, this problem comes from the fact that the loop gain seen by the residue displacement is different with that seen by the quantization noise, and it is far less than the latter. We point out that enhancing of the loop gain is the key to solving this problem, but this compromises loop stability. Furthermore, the chip has integrated a digital BIST function aimed for on-chip dynamic non-linearity analysis. The proposed BIST method utilizes the ΣΔ nature of the interface. An electrical-only single bit signal is used as the BIST stimulus, which is ΣΔ modulated too, thus, the noise and linearity performance of the stimulus is easily assured. The use of 1-bit signal has also alleviated the need for a digital multiplier, resulting in area-efficient implementation, satisfying the requirement of an on-chip harmonic test. The BIST results show that the harmonic distortion can be effectively suppressed by enhancing the loop gain when scaling of the feedback voltage, and the minimum detectable 3-order distortion can be as low as −110 dB.

Although we have provided a glimpse of digital BIST method for EM-SD accelerometer, the on-chip test of the critical parameters (such as scale factor and bias) are left unsolved. This is due to the fact that those parameters are absolute values, but the practical relationship between the electrical stimulus and physical correspondent is hard to get, and will change with the environment variation. Whereas the harmonic distortion test is a relative test, minor variation in amplitude will not affect the test results. Thus, at present, only harmonic distortion is tested by the proposed BIST method. However, due to the flexibility of the proposed BIST method, different test mechanisms could be exploited. As the test results from various angles are collected, the other parameters may be predicted by a comprehensive analysis. This is a future object of this work.

## References

[1] Zwahlen P., Dong Y., Nguyen A.M., Rudolf F., Stauffer J.M., Ullah P., Ragot V. Breakthrough in high performance inertial navigation grade sigma-delta MEMS accelerometer. Proceedings of the 2012 IEEE/ION Position, Location and Navigation Symposium.

[2] Petkov V.P., Boser B.E. (2006). High-order electromechanical ΣΔ modulation in micromachined inertial sensors. IEEE Trans. Circuits Syst. I Regul. Pap..

[3] Jiangfeng W., Carley L.R. (2006). Electromechanical ΔΣ modulation with high-Q micromechanical accelerometers and pulse density modulated force feedback. IEEE Trans. Circuits Syst. I Regul. Pap..

[4] Kulah H., Chae J., Yazdi N., Najafi K. (2006). Noise analysis and characterization of a sigma-delta capacitive microaccelerometer. IEEE J. Solid State Circuits.

[5] Chen F., Li X., Kraft M. (2016). Electromechanical sigma delta modulators force feedback interfaces for capacitive MEMS inertial sensors: A review. IEEE Sens. J..

[6] Zhao M., Chen Z., Lu W., Zhang Y., Niu Y., Chen G. (2017). A high-voltage closed-loop SC interface for a ±50 g capacitive micro-accelerometer with 112.4 dB dynamic range. IEEE Trans. Circuits Syst. I Regul. Pap..

[7] Chen F., Chang H., Yuan W., Wilcock R., Kraft M. (2012). Parameter optimization for a high-order band-pass continuous-time sigma-delta modulator MEMS gyroscope using a genetic algorithm approach. J. Micromech. Microeng..

[8] Raman J., Rombouts P., Weyten L. (2008). An unconstrained architecture for systematic design of higher order ΣΔ force-feedback loops. IEEE Trans. Circuits Syst. I Regul. Pap..

[9] Xu H., Liu X., Yin L. (2015). A closed-loop ΣΔ interface for a high-Q micromechanical capacitive accelerometer with 200 Ng/$\sqrt{\mathrm{Hz}}$ input noise density. IEEE J. Solid State Circuits.

[10] Stauffer J.M., Dietrich O., Dutoit B. RS9000, a novel MEMS accelerometer family for mil/aerospace and safety critical applications. Proceedings of the IEEE/ION Position, Location and Navigation Symposium.

[11] Shkel A.M. Precision navigation and timing enabled by microtechnology: Are we there yet?. Proceedings of the 2010 IEEE Sensors.

[12] Yazdi N., Ayazi F., Najafi K. (1998). Micromachined inertial sensors. Proc. IEEE.

[13] Lutwak R. Micro-technology for positioning, navigation, and timing towards PNT everywhere and always. Proceedings of the 2014 International Symposium on Inertial Sensors and Systems (ISISS).

[14] Hung S.F., Hong H.C. (2014). A fully integrated BIST ΔΣ ADC using the in-phase and quadrature waves fitting procedure. IEEE Trans. Instrum. Meas..

[15] Hong H.C., Su F.Y., Hung S.F. (2010). A fully integrated built-in self-test Σ-Δ ADC based on the modified controlled sine-wave fitting procedure. IEEE Trans. Instrum. Meas..

[16] Dianat A., Attaran A., Rashidzadeh R., Muscedere R. Resonant-based test method for MEMS devices. Proceedings of the 2014 21st IEEE International Conference on Electronics, Circuits and Systems (ICECS).

[17] Dianat A., Attaran A., Rashidzadeh R. Test method for capacitive MEMS devices utilizing pierce oscillator. Proceedings of the 2015 IEEE International Symposium on Circuits and Systems (ISCAS).

[18] Basith I.I., Kandalaft N., Rashidzadeh R., Ahmadi M. (2013). Charge-controlled readout and BIST circuit for MEMS sensors. IEEE Trans. Comput. Aided Des. Integr. Circuits Syst..

[19] Deb N., Blanton R.D. (2006). Built-in self-test of MEMS accelerometers. J. Microelectromech. Syst..

[20] Variyam P.N., Cherubal S., Chatterjee A. (2002). Prediction of analog performance parameters using fast transient testing. IEEE Trans. Comput. Aided Des. Integr. Circuits Syst..

[21] Natarajan V., Bhattacharya S., Chatterjee A. Alternate Electrical Tests for Extracting Mechanical Parameters of Mems Accelerometer Sensors. Proceedings of the 24th IEEE VLSI Test Symposium.

[22] Dumas N., Azais F., Mailly F., Nouet P. A method for electrical calibration of MEMS accelerometers through multivariate regression. Proceedings of the 2009 IEEE 15th International Mixed-Signals, Sensors, and Systems Test Workshop.

[23] Ozel M.K., Cheperak M., Dar T., Kiaei S., Bakkaloglu B., Ozev S. (2017). An electrical-stimulus-only BIST IC for capacitive MEMS accelerometer sensitivity characterization. IEEE Sens. J..

[24] Glueck M., Oshinubi D., Schopp P., Manoli Y. (2014). Real-time autocalibration of MEMS accelerometers. IEEE Trans. Instrum. Meas..

[25] Frosio I., Pedersini F., Borghese N.A. (2012). Autocalibration of triaxial MEMS accelerometers with automatic sensor model selection. IEEE Sens. J..

[26] Glueck M., Buhmann A., Manoli Y. Autocalibration of MEMS accelerometers. Proceedings of the 2012 IEEE International Instrumentation and Measurement Technology Conference Proceedings.

[27] Rohac J., Sipos M., Simanek J. (2015). Calibration of low-cost triaxial inertial sensors. IEEE Instrum. Meas. Mag..

[28] Ye L., Guo Y., Su S.W. (2017). An efficient autocalibration method for triaxial accelerometer. IEEE Trans. Instrum. Meas..

[29] Zwahlen P., Balmain D., Habibi S., Etter P., Rudolf F., Brisson R., Ullah P., Ragot V. In Open-loop and closed-loop high-end accelerometer platforms for high demanding applications. Proceedings of the 2016 IEEE/ION Position, Location and Navigation Symposium (PLANS).

[30] Institute of Electrical and Electronics Engineers (IEEE) (2013). IEEE Recommended Practice for Inertial Sensor Test Equipment, Instrumentation, Data Acquisition, and Analysis.

[31] Nessler S., Marx M., Manoli Y. (2018). A self-test on wafer level for a MEMS gyroscope readout based on ΔΣ modulation. IEEE Trans. Circuits Syst. I Regul. Pap..

[32] Ezekwe C.D., Boser B.E. Robust compensation of a force-balanced high-Q gyroscope. Proceedings of the 2008 IEEE Sensors.

[33] Kraft M., Lewis C., Hesketh T., Szymkowiak S. (1998). A novel micromachined accelerometer capacitive interface. Sens. Actuators A Phys..

[34] Balachandran G.K., Petkov V.P., Mayer T., Balslink T. (2016). A 3-Axis gyroscope for electronic stability control with continuous self-test. IEEE J. Solid State Circuits.

[35] Dong X., Yang S., Zhu J., En Y., Huang Q. (2018). Method of measuring the mismatch of parasitic capacitance in MEMS accelerometer based on regulating electrostatic stiffness. Micromachines.

[36] Fan D., Liu Y., Han F., Dong J. (2012). Identification and adjustment of the position and attitude for the electrostatic accelerometer’s proof mass. Sens. Actuators A Phys..

[37] Zhou W., Yu H., Zeng J., Peng B., Zeng Z., He X., Liu Y. (2016). Improving the dynamic performance of capacitive micro-accelerometer through electrical damping. Microsyst. Technol..

[38] Seeger J.I., Crary S.B. In Stabilization of electrostatically actuated mechanical devices. Proceedings of the International Solid State Sensors and Actuators Conference (Transducers ‘97).

[39] Bechtold T., Feng L., Schrag G. (2013). System-Level Modeling of MEMS.

[40] Seeger J.I., Boser B.E. (2003). Charge control of parallel-plate, electrostatic actuators and the tip-in instability. J. Microelectromech. Syst..

[41] Fargas-Marques A., Casals-Terre J., Shkel A.M. (2007). Resonant pull-in condition in parallel-plate electrostatic actuators. J. Microelectromech. Syst..

[42] Rocha L.A., Cretu E., Wolffenbuttel R.F. (2006). Using dynamic voltage drive in a parallel-plate electrostatic actuator for full-gap travel range and positioning. J. Microelectromech. Syst..

[43] Nielson G.N., Barbastathis G. (2006). Dynamic pull-in of parallel-plate and torsional electrostatic MEMS actuators. J. Microelectromech. Syst..

[44] Wolfram H., Dotzel W. Stability analysis of a MEMS acceleration sensor. Proceedings of the 2006 International Conference on Applied Electronics.

[45] Veijola T. Equivalent Circuit Models for Micromechanical Inertial Sensors. https://pdfs.semanticscholar.org/0cf6/7a4357cd4c1635dbdf6bfc116e936474177f.pdf.

[46] Xuesong J. (2003). Capacitive Position-Sensing Interface for Micromachined Inertial Sensors. Ph.D. Thesis.

[47] Veillette B.R., Roberts G.W. (1998). On-chip measurement of the jitter transfer function of charge-pump phase-locked loops. IEEE J. Solid State Circuits.

[48] Lu A.K., Roberts G.W., Johns D.A. (1994). A high-quality analog oscillator using oversampling D/A conversion techniques. IEEE Trans. Circuits Syst. II Analog. Digit. Signal Process..

[49] Richard Schreier G.C.T. (2005). Understanding Delta-Sigma Data Converters.

